# Multidimensional screen exposure and its impact on psychological well-being in toddlers

**DOI:** 10.3389/fpubh.2024.1466541

**Published:** 2024-12-11

**Authors:** Sha Luo, Wenjie Guo, Hao Chen, Yidong Zhu, Guowei Zhu, Yingnan Jia

**Affiliations:** ^1^Xuhui Maternity and Child Healthcare Center, Shanghai, China; ^2^Preventive Medicine and Health Education Department, School of Public Health, Fudan University, Shanghai, China

**Keywords:** screen time (ST), screen content, children’s mental health, psychological problems, strengths and difficulties questionnaire (SDQ)

## Abstract

**Objective:**

Previous studies have indicated a link between screen exposure and children’s mental health, but with the emergence of new screen media and a rise in screen content, uncertainties have grown. Our aim was to investigate the impact of screen use on psychological issues in 2- to 3-year-old children, considering screen time and types of screen media and content.

**Methods:**

This cross-sectional study included participants from Shanghai, China, from February to July 2023. Screen use information was collected from children’s caregivers via online questionnaire. Psychological difficulties of children were reported by parents using the Strengths and Difficulties Questionnaire (SDQ).

**Results:**

Among the interviewed children, 15.9% had an average daily screen time exceeding 1 hour (h). 52.1% of them viewed inappropriate, non-child-directed screen content, 37.6% were mainly exposed to educational content, and 18.9% watched more fast-paced screen content than slow-paced content. Multivariate regression analysis revealed that the use of mobile phones (*β* = 1.16, 95% CI: 0.14, 2.18), virtual reality (VR) devices (*β* = 2.57, 95% CI: 0.62, 4.53) and computers for more than 30 minutes (min) per day (*β* = 2.15, 95% CI: 0.99, 3.30) were related to higher SDQ difficulty scores. Watching more fast-paced (*β* = 1.58, 95% CI: 0.80, 2.35) and more noneducational screen content (*β* = 1.01, 95% CI: 0.35, 1.66) were also associated with increased difficulty scores.

**Conclusion:**

The amount of time spent using computers, mobile phones and VR devices and the proportion of exposure to noneducational content and fast-paced content were significantly associated with psychological problems among 2- to 3-year-old children.

## Introduction

Screen exposure includes a series of electronic screen-based activities, such as watching television (TV), playing video games, using smartphones and tablets (1). With the marked increase in the availability of screen devices worldwide, screen exposure has become a central part of daily life for younger generations (2). Research has shown that the amount of children’s screen use generally exceeds the recommended daily amount (3, 4). For example, the World Health Organization (WHO) recommends no screen time for 1-year-olds and a no more than 1 h screen time for 2-4-year-olds (5). However, a recent meta-analysis of 95 samples (89,163 children) revealed that 75.3% of children younger than 2 years failed to meet the guideline to avoid screen use, and only 35.6% of children aged 2 to 5 years met the guideline of no more than 1 h a day of screen time (6). Another study from Australia showed that the average screen time for children under 2 years old was 14.2 h per week, while for preschool children aged 2–5, it reached 25.9 h (7). In China, the prevalence of children’s screen use is also alarming. A cross-sectional study of preschool children in Shanghai showed that the average daily screen time for 3- to 4-year-old children was 170 min; 78.6% of these children exceeded the recommended 1 hour of screen time per day (8). Another noticeable problem is that the initial age of screen exposure has been dropping over the years (9). This trend of excessive and prevalent screen use in young generations has attracted considerable attention.

Young children are in a critical stage of rapid cognitive, emotional, and social development and are more susceptible to the influences of external stimuli, information and their environment (10). Therefore, researchers are highly concerned about whether screen usage in early childhood will have an impact on children’s psychological well-being. Previous studies have revealed that excessive screen time is associated with various psychological difficulties, including emotional, peer-related, concentration, and behavioral problems among young children (11). Liu et al. reported a nonlinear dose–response relationship between depressive symptoms and the overall amount of screen time among children who used digital media for more than 2 h per day (12). A cohort study revealed that prolonged exposure to electronic screens at 18 months of age can lead to an increase in attention deficit/hyperactivity symptoms and a decrease in prosocial behavior at the age of 3 years (13). In recent years, some research has proposed the need to consider the impact of screen time on various media. There is also consistent evidence that the types of screen media could modify the association between screen time and mental health symptoms (14). However, studies have primarily focused on traditional screens like TVs and smartphones, with limited exploration of newer devices such as tablets and virtual reality tools.

Furthermore, screen content has been identified as another significant factor affecting children’s mental health (15). Research has shown that the relationship between screen content and children’s mental health varies and it may depend on the type of screen content (11). For example, Zimmerman found that children exposed to more entertainment content on television before the age of 3 had a higher risk of developing mental health problems later on (16). And violent screen was associated with higher levels of fear, anxiety, depression and other mental health issues among children (17). A recent study discovered that non-child-directed programs were associated with a higher risk for mental health problems in children aged 3 to 6 years (15). Conversely, high-quality and educational content has been associated with potential benefits for cognitive development, particularly when children engage with such content in the presence of parents (18, 19). In addition, researchers have also suggested that editing pace of screen content could influence children’s psychological and cognitive problems (20–23). The above research underscores the importance of considering screen content in various dimensions when evaluating its impact on children’s mental health. Notably, research on screen content remains insufficient, focusing only on specific types of screen content. And few studies considered the implications of factors such as screen time, screen media type collectively.

Screen use behavior is complex due to variations in the age of users, various screen media and functions, abundant types of screen content and different amounts of screen time. It is crucial to delve into the multipronged relationship between screen exposure and children’s mental health in order to further fill the gap in this field (such as insufficient exploration of the multifaceted nature of screen exposure and relatively limited research on toddlers). Our study aimed to investigate the relationships between various types of screen exposure and mental health problems among children aged 2–3 years.

## Methods

### Study design and participants

This cross-sectional study aimed to explore the association between screen exposure and psychological well-being in 2–3-year-old children. A convenience sampling method was used to select participants for this study. The sample was drawn from children aged 2–3 years who had undergone regular physical examinations at all 13 community health centers in Xuhui District, Shanghai. Participants were recruited from February to July 2023 through flyers at community hospitals, posters in community bulletin boards, recommendations from physicians, and announcements in parent groups on social media platforms. The inclusion criteria for participants were: (1) children aged between 2 and 3 years, and (2) children whose caregivers could complete the questionnaire by themselves.

Caregivers of recruited children were contacted via telephone to obtain informed consent, after which the online questionnaire link was immediately sent to their mobile phones using Wenjuanxing, a commonly used platform for web-based surveys in China. A post-hoc power analysis was conducted for the final sample, yielding a statistical power of 82.45% with a medium effect size (f^2^ = 0.30). The Ethics Committee for Medical Research at the School of Public Health, Fudan University, approved this study (IRB# 2023-11-1088).

### Measures

#### Screen exposure

A self-designed questionnaire was used to assess children’s screen use, focusing on screen time and content. Caregivers reported children’s average daily screen time over the past week, including total screen time and time spent on different devices (TVs, computers, consoles, phones, tablets, VR devices, projectors). Response options were categorized into four classes: (1) 0 min, (2) 1 to 30 min, (3) >30 to 60 min, and (4) >60 min. The Cronbach’s *α* coefficient for the screen time dimension of the questionnaire was 0.747.

For screen content, several pairs of different screen content types were included in our questionnaire: (1) Inappropriate content: Caregivers reported the proportion of adult or violent content their children watched, with options: none, minority, or majority. (2) Educational content: We inquired about the proportion of educational versus noneducational content exposure, with response options ranging from mainly exposed to educational content, similar proportion of both types, to mainly exposed to noneducational content. (3) Pace of content: We asked about exposure to fast-paced (switching every ≤10 s) versus slow-paced content (switching every ≥30 s), with response options ranging from mainly exposed to slow-paced content, similar proportion of both paces, to mainly exposed to fast-paced content. To clarify our screen content categories, we listed specific programs in our questionnaire, enabling respondents to accurately identify the content types their children watched.

### Psychological difficulties

Psychological difficulties were evaluated using the parent version of Strengths and Difficulties Questionnaire (SDQ), a widely used self-administered tool for assessing the mental health of children (24). The Chinese version of the SDQ, introduced in 2000, has demonstrated good reliability and validity with a Cronbach’s *α* coefficient of 0.749 (25). The questionnaire comprises five factors with 25 items: emotional symptoms, conduct problems, hyperactivity/inattention, peer relationship problems, and prosocial behavior, each with five items. Responses are scored as 0, 1, or 2, indicating “not true, ““somewhat true,” and “certainly true,” respectively. Reverse scoring is applied to specific items. The first four factors make up the difficulty subscale, and the total difficulty score is calculated by summing the scores of these 20 items. A higher difficulty score reflects more significant psychological challenges.

### Covariates

Demographic variables such as children’s age, sex, only-child status, and caregiver structure were collected. Only-child status was categorized as having one child in the family (yes) or two or more children in the family (no). Caregiver structure was classified into parental care (mothers and/or fathers, possibly others), nonparental care (other than mothers and fathers, like grandparents or nannies), and multiple care (mothers and/or fathers, as well as grandparents). Additionally, parental educational level and annual household income were reported. Parental education was assessed and categorized into five groups: ≤ middle school, high school, college graduate, and > college. Annual household income was reported in thousands (RMB), categorized as ≤ 100, >100 to 300, >300 to 600, >600 to 1,200, and > 1,200.

As screen exposure is often associated with sedentary behavior, physical activity, and sleep, we also considered the potential effects of these lifestyle-related behaviors. Caregivers reported children’s daily sleep time, sedentary time, and outdoor and indoor physical activity time. Based on WHO guidelines for children under 5, 2-year-olds with less than 11 h of sleep and 3-year-olds with less than 10 h were labeled as insufficient sleep group, while others were sufficient. Physical activity time (outdoor and indoor combined) categorized children as “<180 min per day” or “≥180 min per day” group. Sedentary time was divided based on whether it exceeded 1 h per day, and outdoor activity time categorized as <2 h or ≥ 2 h per day according to the Physical Activity Guidelines for Preschool Children (3–6 years old).

### Statistical analysis

The data were extracted from Wenjuanxing in tabular format, initially structured using Excel, and analyzed utilizing SPSS 26.0. Descriptive statistics were utilized to present the findings, with continuous variables reported as the mean ± standard deviation and categorical variables reported as frequencies (%). To assess differences in the mean difficulty scores across various groups, t tests and one-way ANOVA were conducted.

Furthermore, to examine the impact of screen exposure on the children’s SDQ scores, multivariate linear regression models were established. For total and different types of screen time, the “0 min” group was treated as the reference group. For the three variables related to the screen content dimension, the corresponding reference groups were “no inappropriate content,” “mainly exposed to educational content” and “mainly exposed to slow-paced content.” The associations between SDQ scores and screen exposure (including screen time variables and screen content variables) were first assessed in Model 1. Model 2 was further adjusted for sex, age, parental educational level, annual household income, sleep time and outdoor physical activity time. All the statistical analyses in this study were performed using two-tailed tests, with the significance level set at *α* = 0.05.

## Results

A total of 874 questionnaires were collected in this survey. After excluding participants who did not meet the age criteria, 864 participants with valid questionnaires were included, for an effective response rate of 98.8%. Of the 864 children included, 427 (49.4%) were boys and 437 (50.6%) were girls, with a mean age of 2.32 ± 0.47 years (Table 1).

**Table 1:** Basic information and comparison of difficulty score between groups.

Variable	Total sample [n (%)]	Difficulty score			t/F	*p*-value
Child sex					**2.404**	**0.016**
Male	427(49.4)	10.36	±	4.51		
Female	437(50.6)	9.65	±	4.20		
Child age, y					1.021	0.313
2	591(68.4)	9.90	±	4.30		
3	273(31.6)	10.22	±	4.52		
Caregiver structure					2.129	0.120
Parental care	306(35.4)	10.38	±	4.45		
Non-parental care	220(25.5)	9.60	±	4.31		
Multiple care	338(39.1)	9.92	±	4.33		
Maternal educational level					**3.799**	**0.010**
≤Middle school	44(5.1)	11.73	±	4.59		
High school	148(17.1)	9.89	±	3.87		
College graduate	478(55.3)	10.13	±	4.53		
>College	194(22.5)	9.38	±	4.18		
Paternal educational level					**6.83**	**<0.001**
≤Middle school	41(4.7)	12.10	±	4.70		
High school	160(18.5)	10.53	±	3.94		
College graduate	440(50.9)	10.05	±	4.40		
>College	223(25.8)	9.16	±	4.38		
Annual household income, ¥					**7.336**	**<0.001**
≤100 thousand	41(4.7)	11.78	±	4.32		
>100 to 300 thousand	375(43.4)	10.68	±	4.27		
>300 to 600 thousand	300(34.7)	9.25	±	4.10		
>600 to 1,200 thousand	111(12.8)	9.26	±	4.61		
>1,200 thousand	37(4.3)	9.46	±	5.33		
Only-child status					3.403	0.065
Yes	595(68.9)	10.19	±	4.24		
No	269(31.1)	9.59	±	4.63		
Sleep time					**3.184**	**0.002**
Not up to the standard	101(11.7)	11.30	±	4.57		
Up to the standard	763(88.3)	9.83	±	4.32		
Sedentary time					1.402	0.161
≤1 h	421(48.7)	10.22	±	4.35		
>1 h	443(51.3)	9.80	±	4.39		
Physical activity time					**2.228**	**0.026**
<3 h	20(2.3)	12.15	±	3.84		
≥3 h	844(97.7)	9.95	±	4.37		
Outdoor activity time					**4.337**	**<0.001**
<2 h	226(26.2)	11.08	±	4.45		
≥2 h	638(73.8)	9.62	±	4.29		

Bold values indicate variables that are statistically significant.

Within 1 week before the investigation, only 2.2% of the children had not viewed any type of screen media. Most children were exposed to screens for less than 60 min per day on average, while 15.9% of the children had a greater screen exposure time. TVs and mobile phones were used more frequently than other types of screen media. For screen content, nearly half of the children were only exposed to appropriate screen content, while the remainder watched varying levels of inappropriate content. A total of 37.6% of the children watched more educational content, 38% watched more noneducation content, and 26.4% watched similar proportions of both types. In addition, 18.9% of the children watched more fast-paced screen content than slow-paced screen content.

The average difficulty score of the included children was 10.00 ± 4.37 points. Compared with girls, boys had a greater average difficulty score (*t* = 2.404, *p* = 0.016). Children whose parents had the highest education had significantly lower scores than the other children (maternal educational level: *p* = 0.010; paternal educational level: *p* < 0.001). There was a significant difference in difficulty scores among the different annual household income groups (*p* < 0.001). Difficulty scores were also related to lifestyle. The scores of the group with sufficient sleep and the group with more than 2 h of outdoor activity per day were significantly lower than their respective counterparts.

Table 2 presents the comparison of children’s difficulty scores by multiple screen time and screen content groups. No statistically significant difference in difficulty scores was found among groups based on total daily screen time. However, screen time for specific types of screen media affected the scores. The most pronounced differences were among the “computer time” (*p* < 0.001) group, followed by the “VR time” (*p* < 0.001) and “mobile phone time” (*p* < 0.001) groups. For screen content, children who watched more educational content had significantly lower scores than did those in the other two comparison groups. Conversely, children who watched more fast-paced content than slow-paced content had significantly higher scores than did other children.

**Table 2:** Screen exposure status and comparison of difficulty score between groups.

Variable	Total sample [n (%)]	Difficulty score			F	*P*-value
Total time					1.89	0.152
0–30 min	507 (58.7)	9.77	±	4.27		
>30 to 60 min	220 (25.5)	10.42	±	4.72		
>60 min	137 (15.8)	10.20	±	4.13		
Mobile phone time					**23.487**	**<0.001**
0 min	249 (28.8)	8.61	±	3.83		
>1 to 30 min	500 (57.9)	10.31	±	4.30		
>30 min	115 (13.3)	11.67	±	4.94		
VR time					**26.545**	**<0.001**
0 min	771 (89.2)	9.65	±	4.17		
>1 to 30 min	62 (7.2)	12.40	±	4.78		
>30 min	31 (3.6)	14.03	±	4.94		
Computer time					**27.519**	**<0.001**
0 min	571 (66.1)	9.37	±	4.03		
>1 to 30 min	193 (22.3)	10.50	±	4.29		
>30 min	100 (11.6)	12.67	±	5.24		
TV time					2.246	0.106
0 min	223 (25.8)	9.84	±	4.12		
>1 to 30 min	395 (45.7)	9.78	±	4.30		
>30 min	246 (28.5)	10.50	±	4.68		
Game time					**16.880**	**<0.001**
0 min	679 (78.6)	9.59	±	4.09		
>1 to 30 min	133 (15.4)	11.11	±	4.72		
>30 min	52 (6.0)	12.58	±	5.54		
Tablet time					**7.896**	**<0.001**
0 min	433 (50.1)	9.44	±	4.19		
>1 to 30 min	310 (35.9)	10.43	±	4.41		
>30 min	121 (14.0)	10.93	±	4.66		
Projector time					**6.692**	**0.001**
0 min	714 (82.6)	9.79	±	4.18		
>1 to 30 min	103 (12.0)	10.59	±	4.94		
>30 min	47 (5.4)	11.98	±	5.28		
Inappropriate content					0.407	0.666
None	414(47.9)	9.89	±	4.30		
Minority	358(41.4)	10.06	±	4.45		
Majority	92(10.7)	10.32	±	4.40		
Educational content					**3.532**	**0.03**
Mainly exposed to educational content	325(37.6)	9.50	±	4.20		
Similar proportions of both types of content	228(26.4)	10.34	±	4.65		
Mainly exposed to noneducational content	311(36.0)	10.29	±	4.30		
Screen pace					**11.273**	**<0.001**
Mainly exposed to slow-paced content	345(39.9)	9.33	±	4.18		
Similar proportion of both paces of content	356(41.2)	10.08	±	4.27		
Mainly exposed to fast-paced content	163(18.9)	11.27	±	4.71		

Bold values indicate variables that are statistically significant.

The results of multiple regression analysis (Table 3) indicated that exposure to certain kinds of screen media and screen content were significantly related to psychological problems in children. After controlling for other variables, the SDQ score of the group using computers for more than 30 min increased by an average of 2.15 points compared to that of the group not using computers. Children who were exposed to VR devices and mobile phones, regardless of whether the exposure duration lasted more than 30 min, had higher scores than did children who were not exposed. The relationship between the proportion of screen content and the score remained significant after adjusting for potential confounders. Viewing more educational screen content than noneducational content was related to lower scores, while viewing more fast-paced screen content than slow-paced content was associated with higher scores. The regression model explained 23% of the variance in psychological difficulties (R^2^ = 0.23), indicating a moderate level of explanatory power.

**Table 3:** Associations between screen exposure and difficulty score.

	Model 1	Model 2
	β(95%CI)	β(95%CI)
Computer time(ref = 0 min)		
>1 to 30 min	0.27(−0.55,1.08)	0.34(−0.46,1.14)
>30 min	2.31(1.13,3.48)**	2.15(0.99,3.30)**
Mobile phone time(ref = 0 min)		
>1 to 30 min	1.13(0.47,1.79)**	1.10(0.45,1.76)**
>30 min	1.59(0.55,2.63)*	1.16(0.14,2.18)*
VR time(ref = 0 min)		
>1 to 30 min	2.71(1.28,4.15)**	2.12(0.69,3.55)*
>30 min	2.87(0.87,4.86)*	2.57(0.62,4.53)*
Game time(ref = 0 min)		
>1 to 30 min	−0.07(−1.07,0.93)	−0.17(−1.15,0.80)
>30 min	−0.30(−1.96,1.37)	−0.23(−1.87,1.41)
Projector time(ref = 0 min)		
>1 to 30 min	−0.63(−1.65,0.39)	−0.31(−1.32,0.69)
>30 min	−0.03(−1.53,1.47)	0.45(−1.02,1.93)
Tablet time(ref = 0 min)		
>1 to 30 min	0.51(−0.15,1.17)	0.47(−0.18,1.12)
>30 min	−0.15(−1.19,0.88)	−0.30(−1.31,0.71)
Total time(ref = 0 min)		
>1 to 30 min	0.17(−0.53,0.87)	0.40(−0.29,1.10)
>30 min	−0.07(−0.91,0.76)	−0.07(−0.89,0.76)
Inappropriate content (ref = none)		
Minority	−0.09(−0.68,0.50)	−0.10(−0.68,0.48)
Majority	−0.05(−1.01,0.91)	0.04(−0.90,0.99)
Educational content (ref = mainly exposed to educational)		
Similar proportion of both types of content	0.82(0.10,1.54)*	0.73(0.02,1.44)*
Mainly exposed to noneducational	0.96(0.29,1.62)*	1.01(0.35,1.66)*
Screen pace (ref = mainly exposed to slow-paced content)		
Similar proportion of both paces of content	0.42(−0.21,1.05)	0.35(−0.27,0.97)
Mainly exposed to fast-paced content	1.58(0.80,2.36)**	1.58(0.80,2.35)**

Model 1: unadjusted model. Model 2: adjusted for sex, age, parents’ educational level, annual household income, sleep time and outdoor physical activity. *p < 0.05; **p < 0.001. Bold values indicate variables that are statistically significant.

## Discussion

Our study is the first to explore how new screen media types (e.g., VR devices, projectors, tablets) and multiple screen content features relate to toddlers’ psychological well-being. Results indicated that using a computer for over 30 min daily, exposure to mobile phones or VR devices, and viewing fast-paced or non-educational content were linked to higher psychological difficulties in 2-3-year-olds, as measured by the SDQ.

These findings align with prior research showing adverse effects of screen use on children’s mental health. However, our study found that total screen time wasn’t significantly associated with difficulty scores. The result suggests that specific screen device usage may have a more significant impact on mental health than overall screen use (26). Similar effects have been observed in several studies (27–29). A systematic review of longitudinal study on relation between screen time and depression highlighted that total screen time has small to very small effects on subsequent depressive symptoms (14). In contrast, computer use and video gaming, but not television viewing, were shown to be connected with more severe depressive symptoms when examining the effects of various types of screens (28). Additionally, excessive social media and internet use have stronger associations with negative outcomes like self-harm, low life satisfaction, and depressive symptoms compared to electronic gaming or TV viewing (29). This underscores the importance of considering the type of screen time rather than just the total duration.

Numerous studies have focused on the health impact of conventional screen media such as televisions (30), computers and mobile phones (31). In our study, we investigated newer media devices such as tablets, game consoles, projectors, and VR devices. Our findings revealed that among these, VR was the only device significantly linked to children’s psychological difficulties. This may be due to the fact that VR often includes games featuring violent or adult themes, with its unique visual effects providing a highly immersive experience. It may impact mental health in complex ways. Limited research exists on the relationship between VR exposure and children’s mental health. While some studies have explored the potential of VR relaxation for adults with mental health conditions (32, 33), its application and effects in children remain unexplored. Additionally, research on how VR impacts cognition, such as studies in rats showing significant cognitive alterations within the brain due to VR exposure (34, 35). However, research addressing the psychological impact of VR on human users is still limited, especially for young children. This suggests an urgent need to pay attention to children’s use of VR devices and to increase research on the health effects and mechanisms.

In recent years, the diversity of screen content has increased sharply. The surge in various TV programs, applications, video games and user-generated content on social media has made monitoring children’s screen content more complex (19). Previous studies have demonstrated varying associations between screen content and children’s mental health (26). For instance, educational content has been linked to positive psychosocial benefits for preschoolers, particularly in language and literacy development (36), while exposure to violent or adult content has been associated with harmful effects on children’s mental health (17). Our findings aligned with previous research, showing that higher exposure to educational programs was associated with a lower risk of mental health issues. However, we did not find a significant negative effect of exposure to inappropriate content, which may be partly due to caregivers’ limited awareness of their children’s screen content and difficulties in determining its appropriateness.

Most notably, our findings revealed that exposure to more fast-paced screen content than slow-paced content was related to a greater risk of psychological difficulties. While previous research on the impact of content pace on children’s mental health is inconclusive, some studies suggest that fast-paced programs can affect executive function (20–22). The potential mechanism may be that rapid pacing provides less time to reflect on and process the viewed content, potentially leading to cognitive overload and overstimulation of the developing brain (37). Another study demonstrated that viewing fast-paced screen content could trigger dopamine and reward pathways in the brain and is thus associated with attention-deficit/hyperactivity disorder symptoms (27). However, evaluations of the impact of the pace of content were mainly conducted for TV programs or films, leaving a gap in understanding the effects of content pace across various screen media types. Our study’s broad measurement of comprehensive screen content helps address this gap to some extent.

To our knowledge, this study was the first to assess the influence of exposure to new screen media and different screen content on toddlers’ mental health. The results further confirmed that certain screen media like computers, phones, and VR devices had a more pronounced negative impact on children’s well-being. The study also revealed the varying effects of different screen content on children’s mental health, providing insights for regulating screen use.

There are some limitations to this study. First, the use of convenience sampling may limit the generalizability of the findings to broader populations, as participants were not randomly selected. Additionally, children’s screen exposure was measured through caregiver reports, which may introduce surrogate bias and recall bias. To address these issues, we required that questionnaires be completed by the primary caregivers, provided detailed explanations and examples to improve understanding, and limited the recall period to the past week to reduce the cognitive burden. Screen time was classified as a triad variable rather than a precise number, and its measurement was not divided by weekday or weekend, potentially leading to some misclassification of screen time. The questionnaires used were not validated for the Chinese population, affecting data accuracy, and the cross-sectional design does not allow for causal inferences between screen exposure and psychological outcomes. Future research should employ more accurate and objective measurement of screen use and consider longitudinal studies to better establish causal relationships between screen exposure and children’s mental health.

## Conclusion

In conclusion, our study showed that the amount of time spent using computers, mobile phones and VR devices and the proportion of exposure to educational content and fast-paced content were significantly associated with psychological problems among children aged 2–3 years. Our study provides preliminary insights into the potential associations between screen time, the types of screen content, and younger children’s psychological outcomes. Technological innovation in the realm of screen-based behavior monitoring and more longitudinal studies will be essential to further explore the mechanism underlying screen exposure and mental health outcomes.

## Data Availability

The raw data supporting the conclusions of this article will be made available by the authors, without undue reservation.
